# Review: modulation of the oral microbiome by the host to promote ecological balance

**DOI:** 10.1007/s10266-019-00413-x

**Published:** 2019-02-04

**Authors:** Pilar Cornejo Ulloa, Monique H. van der Veen, Bastiaan P. Krom

**Affiliations:** Department of Preventive Dentistry, Academic Centre for Dentistry Amsterdam (ACTA), University of Amsterdam and VU University Amsterdam, G. Mahlerlaan 3004, 1081 LA Amsterdam, The Netherlands

**Keywords:** Host factors, Oral microbiome, Modulation, Symbiosis, Oral health

## Abstract

The indivisible relationship between the human host and its oral microbiome has been shaped throughout the millennia, by facing various changes that have forced the adaptation of oral microorganisms to new environmental conditions. In this constant crosstalk between the human host and its microbiome, a bidirectional relationship has been established. The microorganisms provide the host with functions it cannot perform on its own and at the same time the host provides its microbes with a suitable environment for their growth and development. These host factors can positively affect the microbiome, promoting diversity and balance between different species, resulting in a state of symbiosis and absence of pathology. In contrast, other host factors can negatively influence the composition of the oral microbiome and drive the interaction towards a dysbiotic state, where the balance tilts towards a harmful relationship between the host and its microbiome. The aim of this review is to describe the role host factors play in cultivating and maintaining a healthy oral ecology and discuss mechanisms that can prevent its drift towards dysbiosis.

## Introduction

For millions of years, humans and their oral microbiome have co-evolved in constant interaction [1], creating a combined unit that constitutes biomolecular networks also known as ‘holobionts’ [2, 3]. This symbiotic relationship has been shaped throughout the millennia, influenced by considerable transformations. It has been proposed that the oral microbiota stayed relatively stable until major dietary changes during the history of human evolution, which induced a compositional shift along with a noticeable decrease in its diversity [4]. Moreover, adaptation of bacteria to these new environmental conditions [5, 6] and continuous changes in the environment and lifestyle of the host [7] are believed to have greatly contributed to the present configuration of the oral microbiome in humans.

In this context of mutual and functional integration, a reciprocal and dynamic balance between the human host and its microorganisms determines oral health. This interaction is believed to start very early in the life of an individual. There is evidence of the presence of microbes in the placenta in healthy pregnancies that resemble the microbiome of the oral cavity [8], suggesting an early contact with the oral microbiota of the mother through the blood stream [9]. The co-evolution between the host and its microbiome has succeeded in accomplishing complex biological processes that would not be possible for independent entities. A good example of this is the reduction of dietary nitrate to nitrite by oral bacteria on the tongue. Nitrite is a precursor of nitric oxide (NO), which is a potent vasodilator, that can inhibit platelet aggregation and plays an important role in the regulation of blood pressure [10]. This is supported by the observation that prior oral disinfection using chlorhexidine abolished the blood pressure lowering effects of dietary nitrates [11]. This clearly exemplifies the mutual benefits that result from the maintenance of a balanced oral ecology. This balance can nevertheless, undergo distortions that may lead to a shift from a healthy, symbiotic, relationship to a pathological, dysbiotic one. These distortions can originate from changes in the microbiome as well as changes in the host [12–14]. An example of this would be the bidirectional relationship between diabetes mellitus type 2 and periodontal disease. Evidence shows that diabetes is a risk factor for periodontal disease and at the same time severe periodontal disease increases the severity of diabetes mellitus type 2 [15, 16]. Likewise, periodontal therapy and improved periodontal health have been associated with improved glycaemic control in patients with both diabetes and periodontal disease [17, 18].

Processes involved in maintaining a normal, healthy oral microbiome are yet to be fully understood. Until recently, studies have greatly focused on the healthy and pathological variants of the oral microbiota. Even though host factors have been considered as players in this equation, the focus has been mainly set on the possible harmful effects that host factors could have on the transition towards dysbiosis. The role the host plays in the development and maintenance of a healthy oral microbiome has been somehow overlooked, leaving space for research on its role in cultivating a balanced and healthy oral ecology.

Many of the aforementioned topics have been widely described and it is not the aim of this review to discuss them all. There is extensive literature that addresses these matters that the reader can refer to [14, 19–24]. The objective of this review is to describe the role host factors play in cultivating and maintaining a healthy oral ecology and discuss mechanisms that prevent its drift towards dysbiosis. The key terms for the understanding of this review are defined in Table 1.

**Table 1:** Key terms for the purpose of this review

Term	Definition
Biofilm	Complex community of microbes attached to a surface or to each other, encased in a self-produced extracellular polymeric matrix [25]
Ecological balance	Dynamic equilibrium and harmonious coexistence of organisms and their environment [26]
Dysbiosis	A condition in which the balanced state of the ecosystem is disturbed. These disturbances often correspond to external pressures such as disease states or medications [27]
Homeostasis	Dynamic equilibrium of a biological system understood as its ability to maintain its essential variables constant through the mutual interaction of its components [28]
Intrinsic host factors	Inherent biological characteristics of the host that are in general not intentionally modulated by the host
Extrinsic host factors	Characteristics of the host that are the result of intentional modulation based on external stimuli
Microbiome	The totality of microbes, their genetic information and the environment where they interact [27]
Microbiota	All the microbial organisms that integrate the microbiome [27]
Symbiosis	Two or more species living closely together in a long-term relationship [2]

## Composition of the oral microbiome as a result of an intricate multidirectional network

The development of the oral microbiome and its stability or instability is dependent on a multitude of factors [29]. For the purpose of this review, these will be classified into intrinsic host factors and extrinsic host factors. These will be once more divided into intrinsic factors related to the genetic composition of the host, intrinsic factors related to the oral environment of the host, extrinsic factors modulated by the host and extrinsic factors not intentionally modulated by the host.

**Fig. 1:**
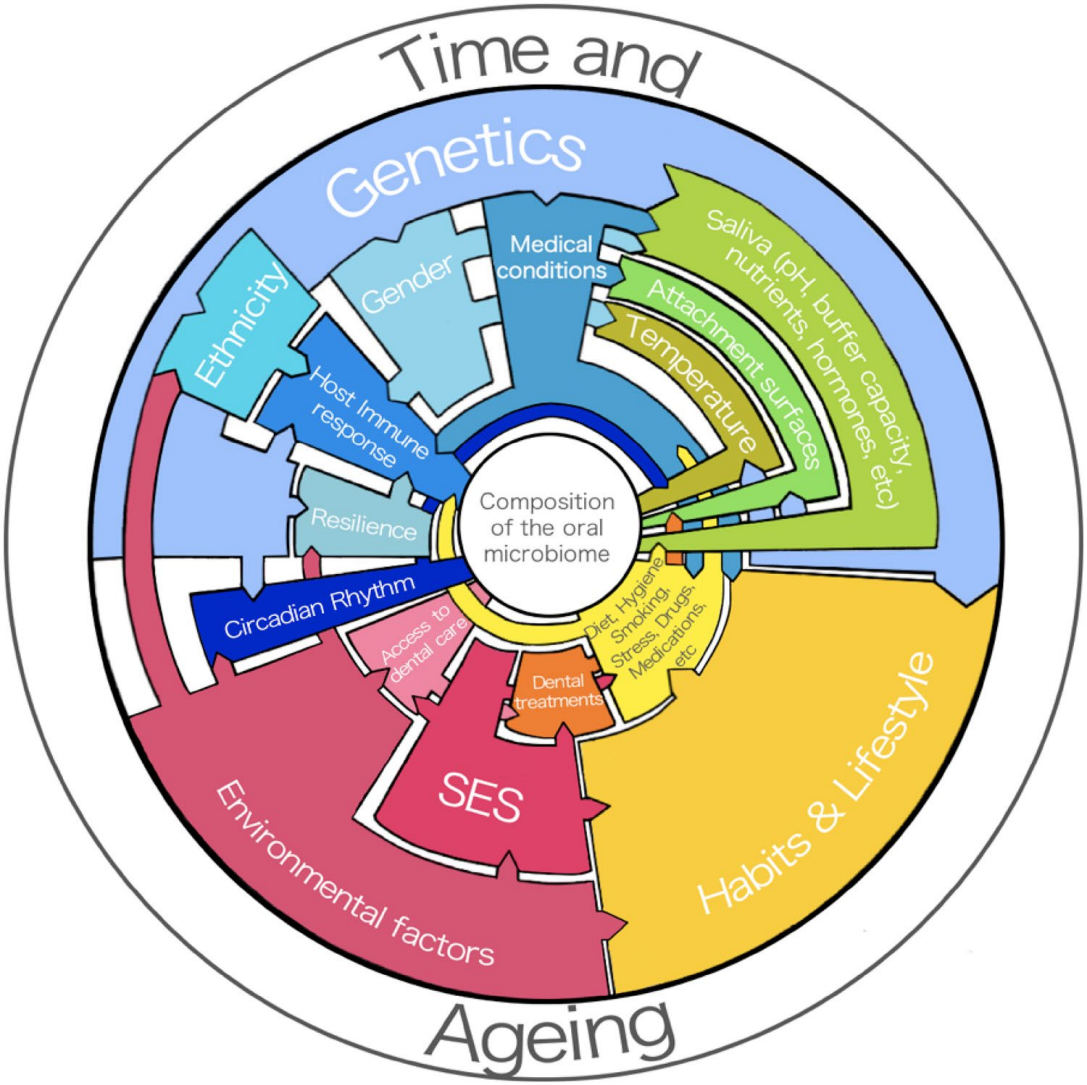
Host factors that can influence the composition of the oral microbiome. Blue: intrinsic factors related to genetics; green: intrinsic factors related to the oral environment; yellow: extrinsic factors modulated by the host; red: extrinsic factors not intentionally modulated by the host; orange: factor with shared characteristics between extrinsic factors modulated by the host and extrinsic factors not intentionally modulated by the host. (Color figure online)

Figure 1 shows the known and recently proposed host factors and their interactions that contribute to development and composition of the human oral microbiome including the physiological changes associated with time and ageing. In other words, these elements are responsible for allowing different microbial species to attach to the oral surfaces, grow and mature. Some of these factors have been found to drive the oral ecology towards a state of dysbiosis. Others, under the right circumstances, will encourage the formation of a balanced and healthy oral ecology and these factors will be discussed below.

## Host–microbiome interactions in health: intrinsic host factors

Intrinsic host factors are defined here as inherent biological characteristics of the host that are in general not intentionally modulated by the host. These factors have a significant presence in the oral cavity and as such exert a direct or an indirect effect on the oral microbiota. They can be further divided into intrinsic factors related to the genetic composition of the host and intrinsic host factors related to the oral environment of the host.

### Intrinsic host factors related to the genetic composition of the host

#### Genetics

There is increasing evidence linking genetic characteristics of the host to the composition of saliva [30], the physical properties of the oral hard tissues [31], the host’s behaviour (i.e., taste perception that influences dietary habits) [32], the host’s immune response [33] as well as the composition of the microbiome across different anatomical sites in the oral cavity [34, 35]. Figure 1 summarises these relations.

Even though the mechanism of first colonization of the oral cavity and shaping of microbial communities is highly influenced by perinatal and possibly prenatal processes [9], genetic host factors play a role in this complex process [29]. Recently, a study that analysed dental plaque from 485 monozygotic and dizygotic twins between ages 5 and 11 showed that there is a heritable fraction of the oral microbiome [36].

Along the same line, ethnicity has been documented to influence the composition of the oral microbiome. It has been hypothesized that the ethnic background of an individual could contribute to a genetic predisposal for the colonization of specific bacteria [37]. Nevertheless, environmental factors such as the country of residence and acculturation (the process by which immigrants adapt to a new culture through changes in beliefs and behaviours) have been reported to influence the ethnical predisposition for certain oral bacteria, changing the oral environment [38, 39]. Even though the exact mechanisms linking the genetic background of the host to the composition of the microbiome are not fully understood yet, there is evidence that suggests that it could be related to variations in the local immune response [29].

#### The host immune response

The oral cavity has a number of defence mechanisms that allow a balanced relationship between the resident microbes and the host. Complex and coordinated innate and adaptive responses operate to maintain the homeostasis between the host and its resident microbes and preserve the physiological functions of the oral cavity [40]. The immune system in health is capable of successfully tolerating the commensal microflora while staying alert for microbes that could pose a threat to the balanced oral ecology. This prevents the occurrence of constant acute inflammatory and allergic reactions despite the great microbial load that colonizes the oral surfaces [41, 42]. Additionally, constant desquamation of oral mucosal surfaces greatly contributes to the control of the bacterial load in the oral cavity [29].

The host immune response is an active process mediated by the coaction of the innate and adaptive immunity of the host [29, 33] and can be affected by several intrinsic and extrinsic factors such as gender [43] or medical conditions [18] among others (See Fig. 1).

##### Innate immunity

Innate immunity protects the host against threats present in the environment [44]. The mucosal surfaces and the enamel protect the deeper tissues of the oral cavity and the teeth from bacterial invasion. These physical barriers along with saliva, transmigrating polymorphonuclear leukocytes (PMNs) and gingival crevicular fluid (GCF) compose the first lines of defence against the microbes present in the biofilm [14, 44]. Antimicrobial peptides, continuously secreted in saliva and gingival crevicular fluid (GCF) protect the periodontium and the oral hard tissues against pathogens [14]. These antimicrobial peptides are also secreted by mucosal epithelial cells and neutrophils, helping to further protect the host against pathogens [45] as well as aiding removal of microorganisms by swallowing posterior to the desquamation of the mucosal surfaces [29, 46]. Mucins, lysozyme, cystatins, defensins and histatins are some of the molecules secreted by the host that regulate the oral microbiota [29]. These defence mechanisms, although fast in its onset, can only provide short-term protection and have a limited specificity [44].

##### Adaptive immunity

The adaptive or acquired immunity is a highly specific immune response that is stimulated by both pathogens and the innate immune response they activate [47]. In the oral mucosa, intraepithelial lymphocytes, Langerhans cells and immunoglobulin A (IgA) and G (IgG) perform this function. Other adaptive immunity components such as IgM and complement are secreted mainly from GCF and are therefore also found in saliva [48]. Secretory IgA is the main salivary immunoglobulin found in health and is capable of inhibiting attachment of bacteria to soft and hard tissues of the oral cavity through different mechanisms [49]. Secretory IgA has the ability to neutralize antigens before they can cause an inflammatory response by a process called immune exclusion. In this process, IgA binds to a wide range of bacteria and viruses, impeding their interaction with oral surfaces benefitting oral health [50]. Thus, innate and adaptive immunity can actively modulate the microbial composition in the healthy oral cavity (Fig. 1).

#### Circadian rhythm

The circadian rhythm is controlled by genetic factors as well as environmental factors [51], as shown in Fig. 1. In this regard, a relationship between the salivary metabolome and our biological clock has been proposed. A study linking the human metabolome and circadian oscillations found that ~ 15% of the identified metabolites in plasma and saliva were controlled by the host’s endogenous circadian rhythm and therefore independent of sleep or feeding [52]. In line with this premise, recent studies have tested the influence of daily rhythms on the oral microbiome [53, 54]. Takayasu et al. tested the influence of the circadian rhythm on the oral microbiota using salivary samples taken periodically, every 4 h, for a period of 3 consecutive days, documenting a significant periodical oscillation of certain phyla. They concluded that certain salivary microbes are active in the daytime (mainly genera *Prevotella*), whereas others metabolize salivary products in the evening (mainly genera *Streptococcus*), suggesting that the circadian rhythm could influence the metabolic activity of the oral microbiome. On the other hand, Collado et al. analysed the impact timing of food intake could have on the composition of the oral microbiome. They showed that food timing could influence the daily rhythms of the salivary microbiota in diversity and abundance. Despite the potential relevance of these outcomes, the number of study subjects was very limited and these results must be addressed with caution. Only recently, circadian rhythm has been proposed as a possible modulating factor of the oral microbiome and the amount of supportive evidence for this claim is still scarce. More research on this topic is needed to further elucidate the mechanisms that could govern this complex process.

#### Resilience of the host

The oral environment can be driven into a state of dysbiosis under certain circumstances [13]. Nevertheless, different hosts can differ greatly in their responses to disease drivers and it has been hypothesized that this may be linked to the resilience capacities of each individual [23]. The concept of resilience can be defined as the ability of the host to recover from a diseased state [55]. Some hosts are more tolerant to disturbances in their environment and more capable of returning to their original balanced state sooner in comparison with a less resilient host [56].

Adaptive psychosocial characteristics have been assessed in search for coping mechanisms that could help the host deal with stressors. Chen et al. investigated the underlying mechanisms associated with adaptive mechanisms to cope with environmental stressors (termed shift-and-persist) [57]. By measuring the socioeconomic status (SES), perception of stress through a questionnaire and inflammatory response to microbial stimulation, they concluded that subjects with a lower SES—which are more exposed to external stressful factors than subjects with a higher SES—had a lower inflammatory response to microbial stimulation and higher levels of shift-and-persist in comparison with their higher SES counterparts [57]. This suggests that psychosocial mechanisms can induce a different biological response that could contribute to the host’s resistance and recovery from disease. In their review of the resilience of the oral microbiome in health, Rosier et al. [23] have proposed that certain health-maintaining mechanisms are enhanced in tolerant individuals. These mechanisms can be at the microbial and host level and could prevent the drift towards a dysbiotic state when present, promoting resilience.

### Intrinsic host factors related to the oral environment

#### Saliva

Saliva is crucial in the maintenance of oral health. It serves an important number of functions in the oral cavity that range from lubrication, cleansing and protection of the oral tissues, to the articulation of speech. Moreover, saliva allows the formation of the acquired pellicle on hard surfaces of the oral cavity, which has an essential role in initial adhesion, colonization and composition of the resident oral microbiota [58]. Lastly, saliva provides a continuous source of nutrients and signalling molecules to the oral microbiota, providing the host a means to influence its symbionts (Fig. 1).

#### Nutritional factors that influence the composition of the microbiome

Saliva is the medium by which the host supplies its resident microorganisms with what they need to survive, e.g., nutrients and trace elements. These nutrients include amino acids, proteins, glycoproteins, peptides and vitamins. In a smaller proportion, the gingival crevice, through GCF secretion, contributes with additional nutrients such as albumin and other glycoproteins and, perhaps more importantly, heme-containing molecules as a source of vital iron [21].

Host hormones delivered through saliva can also be utilized as nutrients by resident bacteria [59, 60]. It has been documented that *Actinobacillus actinomycetemcomitans* and *Porphyromonas gingivalis* are capable of reducing testosterone to 5α-dihydrotestosterone [61]. Moreover, in vitro metabolism of cholesterol and sex steroid hormones by *Treponema denticola* has been documented. Clark and Soory observed that cholesterol is capable of inducing growth of *Treponema denticola*, whereas high concentrations of sex hormones inhibit growth [62]. Furthermore, sex steroid hormones can have an effect on bacterial metabolism, growth and expression of virulence factors [60].

Catecholamines (i.e., epinephrine, norepinephrine, dopamine) are hormones synthesised in the adrenal medulla that are released during a stress response [63]. In saliva, catecholamines have been documented to influence the growth of periodontal bacteria in vitro, having a growth stimulatory or inhibitory effect, depending on the bacterial species. This result suggests that these hormones have the potential to modulate the composition of the oral microbiome [64, 65]. Hence, saliva is the medium that provides a great variety of host molecules that affect the oral microbiome.

#### Salivary cortisol

Cortisol is a steroid hormone that is secreted by the adrenal cortex of the adrenal gland [66]. Synthesis of this hormone follows the circadian rhythm but can also increase in response to a stressor [67]. A number of studies have observed an association between increased cortisol levels in saliva and a higher caries experience in children, suggesting a modulating effect on the oral microbiome [68–71]. The proposed mechanism explaining this relation is based on evidence that chronic presence of cortisol in saliva can impair the function of IgA and affect the immunological response of the host [72–74]. Nevertheless, Tikhonova et al. recently published a review where they concluded that the available studies cannot provide robust evidence due to methodological flaws such as sampling methods and lack of follow-ups [75]. Thus, the relationship between increased salivary cortisol and the composition of the microbiome is still poorly understood.

#### Buffer capacity and salivary pH

The buffering capacity of saliva is responsible for maintaining a favourable pH in the oral cavity (between 6.75 and 7.25), which provides a large number of species of the oral microflora with a suitable environment for growth and  enabling microbial diversity to be maintained [29, 46]. A low buffer capacity modulates the biofilm by exposing it to prolonged periods of low pH resulting in selective growth of more acidogenic bacteria [76]. This results in loss of diversity and represents a dysbiotic state ultimately leading to caries.

Differences in salivary pH have been associated with gender. It has been observed that women present a lower salivary pH than men. Some of the attributed causes for this disparity are related to physiological gender differences. The influence of sex hormones, different biochemical salivary profiles, smaller salivary glands in women and differences in gene expression could explain this variance [77]. Dietary protein intake has also been suggested to induce changes in the salivary pH, linking higher values to higher intake [78]. According to the available evidence, salivary pH is influenced by gender but can also be modified by the host’s dietary habits (Fig. 1).

#### Attachment surfaces

During the course of life, the oral cavity undergoes changes that allow bacteria to colonize different sites. In infants, the only available surfaces for attachment are the mucosa and the tongue. Adhesion to these surfaces by pioneer microorganisms has been documented to occur as early as 10 min after birth [79]. Subsequently, the onset of teeth eruption at around 6–8 months of age provides new surfaces that serve as unique ecological niches that favour further colonization [80]. Changes in the oral environment upon incorporation of restorative materials [81, 82], orthodontic fixed appliances [83], implants [84] or dentures [85, 86] can further modify the ecology of the oral cavity and have consequences on the oral microbiome. Presence of artificial materials in the oral cavity cannot be strictly classified in one group. This factor shares common characteristics with the groups (1) extrinsic factors modulated by the host and (2) extrinsic factors not intentionally modulated by the host. For this reason, it has been located between these two groups (see Fig. 1).

#### Temperature

The temperature of the body, an intrinsic host factor regulated by the nervous system [87], is also influenced by gender [88] as shown in Fig. 1. The human mouth is kept at a reasonably constant temperature between 34 and 36 °C. This condition allows for growth and development of a broad range of microorganisms [89]. Sudden changes in temperature, during hot or cold food and liquid intake, return to baseline levels after some minutes [90, 91]. Nevertheless, prolonged changes in physiologic oral temperatures can induce differential expression of functional genes in oral bacteria [92] which could eventually favour the competitiveness of individual species. Differences in temperature and its relation to inflammation have been studied. It has been observed that subgingival sites with active disease present a higher temperature in comparison to healthy sites [93, 94]. Subsequently, this rise in temperature has been associated with the presence of pathogenic bacteria in the affected sites [94, 95]. Thus, while short changes in temperature will not have major impact on the composition of the oral microbiome, sustained periods of temperature changes as a consequence of inflammation will cause environmental changes that will benefit certain species over others. This could eventually tilt the balance towards promotion of certain pathogenic microorganisms and the detriment of other species.

## Host–microbiome interactions in health: extrinsic host factors

Extrinsic host factors refer to external stimuli that have the potential to affect an individual and can exert an influence in its host characteristics. These factors are not inherent biological characteristics of the host and can be further divided into modulated by the host (lifestyle and behaviour) and not intentionally modulated by the host (environmental factors).

### Extrinsic host factors modulated by the host

#### Lifestyle and behaviour

Dynamic crosstalk between the host and its microbiome is highly influenced by aspects of modern lifestyle and behaviour. Dietary preferences, consumption of substances—such as tobacco or drugs—oral hygiene practices, amongst others, can have an important influence in the composition of the oral microbiome (Fig. 1). Moreover, the presence or absence of these factors can either favour the destabilization of the oral environment and lead to a diseased state or promote ecological balance [96]. Obesity has been associated with higher incidence of dental caries [97] and periodontal disease [98, 99]. Moreover, cigarette smoking and alcohol consumption have also been associated with periodontitis [100]. The effects lifestyle and behaviour can exert on the composition of the oral microbiome have been previously discussed [20, 101, 102] and this review will only focus on diet and oral hygiene.

#### Diet

The frequent consumption of fermentable carbohydrates is an important—yet not the only—cause for developing dental caries by driving the plaque ecology towards a state of dysbiosis with the host [20, 29, 103]. These dietary carbohydrates are fermented by the microbiota into organic acids. If the buffer capacity of saliva is exceeded and these acids are not neutralized, the local pH of the oral environment will drop. This will give a selective advantage to acid-producing and acid-tolerant species and—if sustained in time—will drive the oral ecosystem towards dysbiosis, characterized by more carbohydrate fermenting bacteria that are capable of growing and developing at a lower pH [104].

Diet has also been found to influence the development of periodontal disease. Micronutrient deficiencies such as vitamin C, vitamin D, antioxidants, lower docosahexaenoic acid intake and lower serum magnesium/calcium levels positively correlate with higher levels of periodontal disease [105].

So far, studies assessing modulating effects diet could have on the microbiome have been mostly addressed from a pathological perspective. Dietary fermentable carbohydrates have been clearly identified as gravitating agents in the pathogenesis of disease. Nevertheless, the effect certain dietary components exert on the oral microbiota remains poorly understood. Considering that dietary intake directly influences endogenous nutrients present in the oral cavity through systemic circulation [106], it is not unrealistic to consider that dietary intake may play a role in influencing the composition of the oral microbiome.

Associations between dietary consumption and other environmental factors have been considered. Belstrøm et al. analysed saliva samples from 292 individuals with low levels of oral disease in search for a relation to diet, lifestyle and socioeconomic status (SES) [107]. They found that smoking (lifestyle) and SES were reflected in the composition of the oral microbiome unlike diet that showed no significant relation to the oral bacteria profiles of the study subjects in their sample.

Moreover, differences between dietary intake and specific types of diets such as carnivore, omnivore and vegan have been studied. In 2014, the microbial diversity of 161 healthy individuals that followed an omnivore or ovo-lacto vegetarian or vegan diet was analysed. The results did not show an influence of the dietary choices of the participants in the composition of the microbiome [108]. However, a recent study of 160 participants that followed an omnivore diet or a vegan diet did show differences in the microbiome composition. These variances were attributed to the ingestion of specific macro- and micronutrients [109]. Currently, the evidence is not conclusive and there is no consensus whether these types of diets have a modulating effect on the oral microbiota.

Associations between the oral microbiome and dietary intake (not related to a specific type of diet) have also been assessed. In a recent exploratory study, a modest relationship between the oral microbiome and the dietary intake of 182 study subjects was found [106].

Furthermore, different dietary components have been studied in search for modulatory effects on the oral microbiome. The influence of short-, medium- and long-chain fatty acids on the oral bacteria and fungi has been investigated and inhibitory properties have been described, suggesting a modulating effect on the oral ecology [110].

So far, the available evidence to assess the real impact of dietary preferences and different nutrients on the oral microbiome is still insufficient and more studies are needed.

#### Oral hygiene

Certain behaviours including frequent consumption of fermentable carbohydrates coupled to inadequate fluoride exposure and poor oral hygiene will greatly increase the risk of a drift in the oral ecology towards a state of disease [103].

It has been established that regular day brushing to mechanically remove dental plaque is important for maintaining a healthy oral ecology [111]. Brushing along with the use of fluoride has been clinically proven to be significantly more beneficial than mechanical plaque removal alone [112]. Fluoride has been pointed as the main factor responsible for the rapid decline in caries prevalence in the world [113]. The mechanisms of action of fluoride have been already explained in detail [114] and are beyond the scope of this review. In general, fluoride interferes with the process of dental caries formation by conferring protection to the enamel on its outer layer as well as in its inner structure, making it more resistant to acid attack. Topical action of fluoride has been proved to be more effective than incorporation of fluoride in the inner structure of the tooth during its formation [115]. Fluoride’s possible antimicrobial effects on the oral biofilm have been investigated in vitro and in vivo. In vitro studies have shown that fluoride could have an influence on important bacterial biological processes such as enzyme inhibition and acid production of certain species such as *Streptococcus mutans* [116, 117]. Studies in vivo have observed a reduction in plaque formation that could be influenced by the concentration of fluoride used in toothpaste [116]. Nevertheless, the existing evidence is not conclusive and the direct effects that fluoride could have on the oral microbiome are yet to be elucidated.

Oral hygiene must be compatible with health. In other words, bacterial mechanisms that drive the oral ecology to a state of dysbiosis must be controlled without damaging beneficial bacteria [118] which perform several important functions at the host level [96]. Considering that a state of ecological balance is not compatible with the eradication of the oral microbiome, the promotion of a balanced oral ecology should focus first on the control of factors that can induce a drift towards a dysbiotic state (e.g., reducing the intake and frequency of fermentable carbohydrates). In case oral hygiene products are needed, these should work at sub-lethal concentrations to prevent a drift towards a dysbiotic state and at the same time keep the beneficial bacteria alive and capable of continuing their vital activities [119]. From this perspective, oral ecology-modulating strategies are preferred over the use of antimicrobials.

### Extrinsic host factors not intentionally modulated by the host

#### Environmental factors

These factors are determined by the place of birth of the host and the (cultural) conditions that are present at the place of domicile. In general, these factors can only occasionally be changed.

#### Socioeconomic status (SES)

The relationship between SES and the incidence of oral diseases such as caries and periodontitis has been widely studied [120–123]. Social inequalities in oral health and disease have been mostly associated to income, social position, education and residence area of the studied populations [122]. These factors can in part explain the incidence of disease and its relation to SES from a social point of view (Fig. 1). In an attempt to find a physiological rationale, studies that link SES with differential biological responses have been published. For instance, Boyce et al. assessed the SES of elementary school children, their basal salivary cortisol and the presence of cariogenic bacteria in saliva [69]. They found a strong association between cariogenic bacteria and SES, linking a higher acquisition of cariogenic bacteria to a lower SES. This association increased when higher levels of basal salivary cortisol were present [69] suggesting that external stressor factors associated with a lower SES could promote acquisition of caries associated bacteria. Studies have observed a high correlation between SES and the occurrence of oral disease but the exact biological mechanisms that could explain this phenomenon are yet to be elucidated.

#### Access to dental care

Statistics available for the US show that the level of access individuals have to dental care will depend on: (1) the geographical location and the availability of an oral health professional to deliver dental care and, most importantly, (2) on the financial barriers that could keep a group of individuals from accessing oral health services [124] as shown in Fig. 1. This finding is closely related to SES and should be addressed as a whole. Hence, the implementation of oral disease prevention programmes as well as possibility of access to oral care should have a special focus on addressing the most vulnerable groups of society.

## Conclusion

The bidirectional relationship between the host and its microbiome is extremely dynamic. In this constant process, the microbiome will provide the host with functions it needs to survive. Parallel to this, the host will provide its microbiome with a suitable environment for its growth and development. The sustainability of a balanced relationship between the host and its microbiome is to their mutual benefit.

Many factors can influence the maintenance of this balance in the oral ecology. In other words, a balanced state does not rely on a single factor but is rather the resultant of all interactions, antagonistic and synergistic, of diverse elements in different proportions, which will actively determine the modulation of the oral microbiome, contributing to its composition and behaviour.

These elements are: (1) intrinsic factors inherent to the host and highly influenced by its genetic background and (2) extrinsic factors related to the environment where the host develops and factors related to its lifestyle and behaviour.

The understanding of underlying processes that allow the host to positively influence its oral microbiome remains poorly understood. There is growing evidence that intrinsic factors of the host play a major role in modulation of the oral ecology. This could explain why certain individuals develop different responses to equal stimuli.

Further research in this direction is needed to shed light on individual factors of the host that favour symbiosis with its microbes and can have an impact on the oral ecology, leading the way to more personalized and integrative care in dentistry and oral medicine.
